# *RET* fusion driven (*RETfus+*) non-small cell lung cancer: a comprehensive genomic profiling study with histologic correlation

**DOI:** 10.3389/fonc.2025.1477910

**Published:** 2025-05-16

**Authors:** Prashanth Ashok Kumar, Michael Connolly, Alina Basnet, Dean Pavlick, Richard Huang, Steven Graziano, Jeffrey Ross

**Affiliations:** ^1^ Division of Hematology-Medical Oncology, Upstate Cancer Center, SUNY Upstate Medical University, Syracuse, NY, United States; ^2^ Foundation Medicine, Cambridge, MA, United States; ^3^ Department of Pathology, SUNY Upstate Medical University, Syracuse, NY, United States

**Keywords:** non-small cell lung cancer, RET fusion, targeted therapy, genomic profiling, lung cancer

## Abstract

**Background:**

Fusion of the *RET* gene resulting in clinically significant Genomic Alteration (GA) occur in 1-2% of NSCLC in the United States and has emerged as a major target for *RET* inhibitors which are first line treatment options in the Stage 4 setting. *RET* fusions have also been well-described as acquired resistance mutations in cases of *EGFR*-driven NSCLC treated with anti-*EGFR* tyrosine kinase inhibitors including erlotinib and osimertinib. The aim of this study was to determine whether *RET* fusion positive (*RETfus+*) NSCLC represents a unique histologic subtype of the disease with a unique genomic profile.

**Methods:**

We selected 503 of 72,596 (0.7%) total NSCLC that were reported as *RETfus+* from the Foundation One database. The cases were centrally evaluated for predominant histology and underwent hybrid capture based CGP to evaluate diverse GA. Cases with *EGFR* mutations were excluded. PD-L1 expression was determined by Immunohistochemistry (IHC) (Dako 22C3) with Tumor Proportion Score (TPS) ≥50% = high expression. For statistical comparisons, the false discovery rate was corrected using Benjamini/Hochberg adjustment.

**Results:**

Potentially targetable GAs found less frequently in the *RETfus+* group included *BRCA1, BRAF, FGF12, FGFR1, KEAP1, KMT2D, KRAS, MDM2, MET, NF1, NSD3, PIK3CA, RB1*, AND *TP53*. The presence of *HRD, APOBEC* and *Tobacco* gene signatures were also lower in frequencies in the *RETFus+* NSCLC cases. *SETD2* was the only GA found to be higher in the *RETfus+* group. While markers predictive of checkpoint therapy response including TMB high level was more frequent in the *RETfus-* cases, PD-L1 high expression was more in *RETfus+* samples. Surgical pathology analysis revealed that the high grade solid non-acinar pattern at 32% was the most frequent histologic subtype.

**Conclusions:**

*RETfus+* NSCLC features a unique genomic signature which can further impact therapy selection. With recent expanded approval of more specific RET kinase targeting inhibitors (selpercatinib and Pralsetinib) in the pan-cancer treatment setting, further study of *RETfusion+* NSCLC histology and genomic/biomarker status appears warranted.

## Introduction

Precision medicine has revolutionized the management of clinically advanced and metastatic Non-Small Cell Lung Cancer (mNSCLC), to the extent that it has become standard practice to perform Next Generation Sequencing (NGS) for formulating a treatment strategy (1, 2). NGS has expanded to limited stage disease and there is an evolving spectrum for Targeted agents for various settings (3). Currently, 350 individuals per day die from lung cancer, making it the leading cause of cancer death (4), prompting the need for continued research that will enable better understanding of the molecular drivers of the disease.

*RET* fusion (*RETfus+*) are drivers in the oncogenesis of several malignancies including mNSCLC and thyroid cancers. Gene fusions in the *RET* kinase domain result in the production of constitutively active chimeric *RET* homodimers that result in unchecked and aberrant cellular proliferation. This is usually seen in mNSCLC. Whereas in medullary thyroid cancer, germline, or somatic point mutations of kinase domain result in *RET* kinase activation (5). *RETfus+* is seen in 1-2% of mNSCLCs, Today, patients have oral therapeutic options like selpercatinib and pralisetinib for treatment of this type of NSCLC (6, 7). These agents are relatively well-tolerated and have also demonstrated intracranial activity particularly with selpercatinib (6, 7). Although traditionally believed to be mutually exclusive, the hypothesis that *RETfus+* may be a unique pathologic entity originated with reports showing its association with other alterations and biomarkers. Co-occurrence with *EGFR* mutations, *MET* amplification, low Tumor Mutational Burden (TMB) and PD-L1 expression have also been documented (8). *RETfus+* NSCLC demonstrates increased responsiveness to pemetrexed based chemotherapy (9) and may also have a role in resistance to other targets like *EGFR* (10). Therefore, understanding the unique distribution of genomic alterations (GA) that occurs in *RETfus+* mNSCLC is essential (8, 11).

## Methods

The study was approved, and a waiver of consent was obtained from the Western Institutional Review Board (Protocol No. 20152817) and SUNY Upstate IRB (Project No. 2144270). FoundationOne is a CLIA-certified and CAP-accredited reference molecular laboratory. A database query was done for all mNSCLC cases submitted to Foundation Medicine. All cases were clinically advanced, either inoperable or metastatic. Basic details such as patient age and gender, routine histology, immunohistochemical staining results and confirmation of the diagnosis, were extracted from the pathology reports. All cases were sequenced between January 2018 and July 2022. Cases needed have had adequate tissue sample, DNA amount of 50 ng or greater and minimum of 20% tumor nuclear area versus benign nuclear area either before or after pathologist-guided macro-enrichment. Cases with low tumor purity on sequencing or inadequate sequencing coverage depth as described in the Foundation Medicine US FDA approval were excluded from the analysis (12).

Sample sequencing was done for Comprehensive genomic profiling (CGP) on 72,506 mNSCLC tissue samples. DNA was extracted using hybridization-capture- adaptor ligation–based libraries (FoundationOneCDx, Foundation Medicine, Inc.). Assay was done using all coding exons from 324 cancer related genes, plus select introns from at least 31 genes frequently rearranged in various malignancies. Specimens were evaluated for all classes of GAs including base substitutions, insertions, deletions, copy number alterations (amplifications and homozygous deletions), and for select gene fusions/rearrangements. Bioinformatics processes included Bayesian algorithms to detect base substitutions, local assembly algorithms to detect short insertions and deletions, a comparison with process-matched normal control samples to detect gene copy number alterations and an analysis of chimeric read pairs to identify gene fusions. An oncoprint plot was generated with the online tools of the cbio portal as described by Gao et al (13) and Cerami et al (14). TMB scores were defined by mutation/Mb on 0.83–1.14 Mb of sequence. Assessment of microsatellite instability was performed from DNA sequencing at least 95 loci. Each microsatellite locus had repeat length of 7–39 bp. The next-generation sequencing based “microsatellite instability score” was translated into categorical “microsatellite instability high”, “microsatellite instability intermediate”, or “microsatellite stable” by unsupervised clustering of specimens for which microsatellite instability status was assessed via gold standard methods (15). PD-L1 expression was measured using the DAKO 22C3 assay with low-positive tumor cell scoring defined as 1%-49% staining and high-positive tumor cell scoring defined as >50% staining (16). Anti-PD-L1 staining was done using the Dako 22C3 IHC kit, following the instructions provided in the kit protocol. Results were scored using the widely used tumor proportional score system (TPS) (17). Chi-square test and Mann Whitney U test were used in the statistical comparisons of the 2 groups. Statistical significance was defined as p < 0.05. Odds Ratio (OR) estimates were utilized to study the difference in the likelihood of a GA occurring between *RETfus+* and *RETfus-.* The technique has been described in our previous publications, in which similar templates were followed (18).

## Results

A total of 72,506 mNSCLC patients were identified of which 503 (0.69%) were *RETfus+* and 72,003 (99.31%) were *RET-fusion-*. The age, sex distribution and descriptive analysis of various GAs are depicted in **Table 1**. The mean age was 66 years in *RETfus+* and 69 years in the *RETfus-* group. Sex distribution was similar with 243 (48.31%) males in the *RETfus+* cohort and 35,971 (49.96%) in the *RETfus-* group. The average GA/tumor was 4.6 for *RETfus+* and 6 for *RETfus-.*

**Table 1:** Distribution of various biomarkers among *RETfus*+ and *RETfus*- NSCLC overall and based on histology.

	ALL NSCLC			*RET+EGFR-* Distribution by Histologic subtypes											
N (%)	*RET+EGFR-*	*RET-*	Odds Ratio (OR)/P value	High grade	Non-High grade	OR/P value	Subtype undetermined/Adenocarcinoma, NOS	Non-Subtype undetermined	OR/P value	Papillary	Non-Papillary	OR/P value	Acinar	Non-Acinar	OR/P value
**Total N**	**503**	**72003**		161	342		112	391		102	401		50	453	
**Male sex**	243 (48.31)	35971 (49.96)	0.94/p=0.65	91 (56.52)	152 (44.44)	1.63/p=0.07	48 (42.86)	195 (49.87)	0.75/p=1.00	44 (43.14)	199 (49.63)	0.77/p=0.84	26 (52.00)	217 (47.90)	1.18/p=1.00
**Median age range in years**	66 (28-89) (57-74)	69 (4-89) (62-76)		66 (30-89) (57.75-73)	66 (28-89) (56.5-74)		67.5 (28-89) (55.25-75)	66 (30-89) (58-73)		68 (36-89) (59-74.5)	66 (28-89) (56-74)		65 (37-88) (57.75-73)	66 (28-89) (57-74)	
**GA/tumor**	4.7	6.1													
Genomic alterations															
**Total N**	**503**	**71876**		161	342		112	391		102	401		50	453	
***APC***	9 (1.79)	2137 (2.97)	0.60/p=0.31	3 (1.86)	6 (1.75)	1.06/p=1.00	2 (1.79)	7 (1.79)	1.00/p=1.00	0 (0.00)	9 (2.24)	0.00/p=1.00	1 (2.00)	8 (1.77)	1.14/p=1.00
***ARFRP1***	14 (2.78)	1105 (1.54)	1.84/p=0.13	4 (2.48)	10 (2.92)	0.85/p=1.00	1 (0.89)	13 (3.32)	0.26/p=1.00	5 (4.90)	9 (2.24)	2.25/p=1.00	3 (6.00)	11 (2.43)	2.56/p=1.00
***ATM***	22 (4.37)	3474 (4.83)	0.90/p=0.81	9 (5.59)	13 (3.80)	1.14/p=1.00	4 (3.57)	18 (4.60)	0.73/p=1.00	4 (3.92)	18 (4.49)	0.36/p=1.00	2 (4.00)	20 (4.42)	1.38/p=1.00
***BRAF***	5 (0.99)	3864 (5.38)	0.18/p<0.05	2 (1.24)	3 (0.88)	1.50/p=1.00	1 (0.89)	4 (1.02)	0.77/p=1.00	2 (1.96)	3 (0.75)	0.87/p=1.00	0 (0.00)	5 (1.10)	0.90/p=1.00
***BRCA1***	1 (0.20)	1066 (1.48)	0.13/p<0.05	1 (0.62)	0 (0.00)	1.42/p=1.00	0 (0.00)	1 (0.26)	0.87/p=1.00	0 (0.00)	1 (0.25)	2.65/p=1.00	0 (0.00)	1 (0.22)	0.00/p=1.00
***BRCA2***	7 (1.39)	1467 (2.04)	0.68/p=0.42	1 (0.62)	6 (1.75)	inf/p=0.32	0 (0.00)	7 (1.79)	0.00/p=1.00	3 (2.94)	4 (1.00)	0.00/p=1.00	2 (4.00)	5 (1.10)	0.00/p=1.00
***CCNE1***	7 (1.39)	2324 (3.23)	0.42/p=0.06	3 (1.86)	4 (1.17)	0.35/p=0.44	3 (2.68)	4 (1.02)	0.00/p=0.36	0 (0.00)	7 (1.75)	3.01/p=0.15	0 (0.00)	7 (1.55)	3.73/p=0.15
***CDK4***	27 (5.37)	2391 (3.33)	1.65/p=0.06	11 (6.83)	16 (4.68)	1.60/p=1.00	6 (5.36)	21 (5.37)	2.66/p=1.00	5 (4.90)	22 (5.49)	0.00/p=1.00	2 (4.00)	25 (5.52)	0.00/p=1.00
***CDKN2A***	140 (27.83)	22234 (30.93)	0.86/p=0.31	52 (32.30)	88 (25.73)	1.49/p=1.00	30 (26.79)	110 (28.13)	1.00/p=1.00	16 (15.69)	124 (30.92)	0.89/p=1.00	9 (18.00)	131 (28.92)	0.71/p=1.00
***CDKN2B***	102 (20.28)	13296 (18.50)	1.12/p=0.44	39 (24.22)	63 (18.42)	1.38/p=0.97	22 (19.64)	80 (20.46)	0.93/p=1.00	11 (10.78)	91 (22.69)	0.42/p=0.17	4 (8.00)	98 (21.63)	0.54/p=1.00
***CHEK2***	11 (2.19)	1240 (1.73)	1.28/p=0.50	3 (1.86)	8 (2.34)	1.42/p=0.97	1 (0.89)	10 (2.56)	0.95/p=1.00	2 (1.96)	9 (2.24)	0.41/p=0.36	1 (2.00)	10 (2.21)	0.31/p=1.00
***FGF12***	1 (0.20)	2803 (3.90)	0.05/p<0.05	0 (0.00)	1 (0.29)	2.15/p=1.00	0 (0.00)	1 (0.26)	0.70/p=1.00	0 (0.00)	1 (0.25)	0.00/p=1.00	0 (0.00)	1 (0.22)	1.83/p=1.00
***FGF3***	15 (2.98)	3519 (4.90)	0.60/p=0.14	5 (3.11)	10 (2.92)	0.00/p=1.00	3 (2.68)	12 (3.07)	0.00/p=1.00	3 (2.94)	12 (2.99)	0.00/p=1.00	1 (2.00)	14 (3.09)	0.00/p=1.00
***FGFR1***	4 (0.80)	3189 (4.44)	0.17/p<0.05	0 (0.00)	4 (1.17)	0.00/p=0.97	1 (0.89)	3 (0.77)	1.17/p=1.00	0 (0.00)	4 (1.00)	0.00/p=1.00	1 (2.00)	3 (0.66)	3.06/p=1.00
***FGFR2***	2 (0.40)	378 (0.53)	0.76/p=1.00	0 (0.00)	2 (0.58)	0.00/p=1.00	1 (0.89)	1 (0.26)	3.51/p=1.00	0 (0.00)	2 (0.50)	0.00/p=1.00	0 (0.00)	2 (0.44)	0.00/p=1.00
***FGFR3***	1 (0.20)	661 (0.92)	0.22/p=0.23	0 (0.00)	1 (0.29)	0.00/p=1.00	0 (0.00)	1 (0.26)	0.00/p=1.00	0 (0.00)	1 (0.25)	0.00/p=1.00	0 (0.00)	1 (0.22)	0.00/p=1.00
***KEAP1***	15 (2.98)	9711 (13.51)	0.20/p<0.05	10 (6.21)	5 (1.46)	4.46/p=0.36	1 (0.89)	14 (3.58)	0.24/p=1.00	1 (0.98)	14 (3.49)	0.27/p=1.00	1 (2.00)	14 (3.09)	0.64/p=1.00
***KMT2D***	10 (1.99)	4690 (6.53)	0.29/p<0.05	6 (3.73)	4 (1.17)	3.27/p=0.97	1 (0.89)	9 (2.30)	0.38/p=1.00	1 (0.98)	9 (2.24)	0.43/p=1.00	1 (2.00)	9 (1.99)	1.01/p=1.00
***KRAS***	12 (2.39)	22226 (30.92)	0.05/p<0.05	5 (3.11)	7 (2.05)	1.53/p=1.00	2 (1.79)	10 (2.56)	0.69/p=1.00	1 (0.98)	11 (2.74)	0.35/p=1.00	1 (2.00)	11 (2.43)	0.82/p=1.00
***MDM2***	45 (8.95)	3083 (4.29)	2.20/p<0.05	18 (11.18)	27 (7.89)	1.47/p=0.97	5 (4.46)	40 (10.23)	0.41/p=1.00	10 (9.80)	35 (8.73)	1.14/p=1.00	5 (10.00)	40 (8.83)	1.15/p=1.00
***MET***	6 (1.19)	3772 (5.25)	0.22/p<0.05	4 (2.48)	2 (0.58)	4.33/p=0.97	2 (1.79)	4 (1.02)	1.76/p=1.00	0 (0.00)	6 (1.50)	0.00/p=1.00	0 (0.00)	6 (1.32)	0.00/p=1.00
***MTAP***	80 (15.90)	9948 (13.84)	1.18/p=0.35	30 (18.63)	50 (14.62)	1.34/p=0.97	17 (15.18)	63 (16.11)	0.93/p=1.00	9 (8.82)	71 (17.71)	0.45/p=0.95	4 (8.00)	76 (16.78)	0.43/p=1.00
***NF1***	10 (1.99)	5791 (8.06)	0.23/p<0.05	5 (3.11)	5 (1.46)	2.16/p=0.97	2 (1.79)	8 (2.05)	0.87/p=1.00	0 (0.00)	10 (2.49)	0.00/p=1.00	0 (0.00)	10 (2.21)	0.00/p=1.00
***NF2***	3 (0.60)	1192 (1.66)	0.36/p=0.19	1 (0.62)	2 (0.58)	1.06/p=1.00	0 (0.00)	3 (0.77)	0.00/p=1.00	1 (0.98)	2 (0.50)	1.98/p=1.00	0 (0.00)	3 (0.66)	0.00/p=1.00
***NFE2L2***	13 (2.58)	3427 (4.77)	0.53/p=0.06	4 (2.48)	9 (2.63)	0.94/p=1.00	2 (1.79)	11 (2.81)	0.63/p=1.00	3 (2.94)	10 (2.49)	1.18/p=1.00	0 (0.00)	13 (2.87)	0.00/p=1.00
***NSD3***	5 (0.99)	3583 (4.98)	0.19/p<0.05	0 (0.00)	5 (1.46)	0.00/p=0.97	1 (0.89)	4 (1.02)	0.87/p=1.00	1 (0.98)	4 (1.00)	0.98/p=1.00	1 (2.00)	4 (0.88)	2.29/p=1.00
***PBRM1***	3 (0.60)	1671 (2.32)	0.25/p=0.03	2 (1.24)	1 (0.29)	4.29/p=0.97	0 (0.00)	3 (0.77)	0.00/p=1.00	0 (0.00)	3 (0.75)	0.00/p=1.00	0 (0.00)	3 (0.66)	0.00/p=1.00
***PIK3CA***	13 (2.58)	8231 (11.45)	0.21/p<0.05	5 (3.11)	8 (2.34)	1.34/p=1.00	2 (1.79)	11 (2.81)	0.63/p=1.00	1 (0.98)	12 (2.99)	0.32/p=1.00	2 (4.00)	11 (2.43)	1.67/p=1.00
***PRKCI***	12 (2.39)	3257 (4.53)	0.52/p=0.06	5 (3.11)	7 (2.05)	1.53/p=1.00	0 (0.00)	12 (3.07)	0.00/p=1.00	1 (0.98)	11 (2.74)	0.35/p=1.00	1 (2.00)	11 (2.43)	0.82/p=1.00
***RB1***	20 (3.98)	5988 (8.33)	0.46/p<0.05	6 (3.73)	14 (4.09)	0.91/p=1.00	8 (7.14)	12 (3.07)	2.43/p=1.00	1 (0.98)	19 (4.74)	0.20/p=1.00	4 (8.00)	16 (3.53)	2.38/p=1.00
***RBM10***	3 (0.60)	5176 (7.20)	0.08/p<0.05	2 (1.24)	1 (0.29)	4.29/p=0.97	0 (0.00)	3 (0.77)	0.00/p=1.00	1 (0.98)	2 (0.50)	1.98/p=1.00	0 (0.00)	3 (0.66)	0.00/p=1.00
***SETD2***	54 (10.74)	2078 (2.89)	4.05/p<0.05	12 (7.45)	42 (12.28)	0.58/p=0.97	13 (11.61)	41 (10.49)	1.12/p=1.00	12 (11.76)	42 (10.47)	1.14/p=1.00	7 (14.00)	47 (10.38)	1.41/p=1.00
***SMARC4***	10 (1.99)	5019 (6.98)	0.27/p<0.05	4 (2.48)	6 (1.75)	1.43/p=1.00	2 (1.79)	8 (2.05)	0.87/p=1.00	1 (0.98)	9 (2.24)	0.43/p=1.00	2 (4.00)	8 (1.77)	2.32/p=1.00
***SOX2***	3 (0.60)	4553 (6.33)	0.09/p<0.05	2 (1.24)	1 (0.29)	4.29/p=0.97	0 (0.00)	3 (0.77)	0.00/p=1.00	0 (0.00)	3 (0.75)	0.00/p=1.00	0 (0.00)	3 (0.66)	0.00/p=1.00
***STK11***	12 (2.39)	11641 (16.20)	0.13/p<0.05	6 (3.73)	6 (1.75)	2.17/p=0.97	2 (1.79)	10 (2.56)	0.69/p=1.00	1 (0.98)	11 (2.74)	0.35/p=1.00	1 (2.00)	11 (2.43)	0.82/p=1.00
***TERC***	12 (2.39)	3357 (4.67)	0.50/p=0.05	5 (3.11)	7 (2.05)	1.53/p=1.00	1 (0.89)	11 (2.81)	0.31/p=1.00	0 (0.00)	12 (2.99)	0.00/p=1.00	1 (2.00)	11 (2.43)	0.82/p=1.00
***TP53***	211 (41.95)	50329 (70.02)	0.31/p<0.05	83 (51.55)	128 (37.43)	1.78/p=0.31	49 (43.75)	162 (41.43)	1.10/p=1.00	36 (35.29)	175 (43.64)	0.70/p=1.00	17 (34.00)	194 (42.83)	0.69/p=1.00
***ZNF703***	6 (1.19)	2650 (3.69)	0.32/p<0.05	0 (0.00)	6 (1.75)	0.00/p=0.97	1 (0.89)	5 (1.28)	0.70/p=1.00	1 (0.98)	5 (1.25)	0.78/p=1.00	2 (4.00)	4 (0.88)	4.68/p=1.00
**MSI High**	1 (0.20)	356 (0.49)	0.40/p=0.65	0 (0.00)	1 (0.29)	0.00/p=1.00	0 (0.00)	1 (0.26)	0.00/p=1.00	0 (0.00)	1 (0.25)	0.00/p=1.00	1 (2.00)	0 (0.00)	inf/p=0.38
**N**	**503**	**71997**		161	342		84	300		102	401		50	453	
**Median TMB (Range)**	2.41 (0-45)	6.25 (0-1977.8)		2.5 (0-45)	1.25 (0-36.26)		2.41 (0-15)	2.41 (0-45)		1.25 (0-36.256)	2.41 (0-45)		1.25 (0-27.76)	2.41(0-45)	
**TMB ≥10 mut/Mb**	32 (6.36)	23881 (33.17)	0.14/p<0.05	16 (9.94)	16 (4.68)	2.25/p=0.14	3 (2.68)	29 (7.42)	0.71/p=1.00	5 (4.90)	27 (6.73)	0.71/p=1.00	3 (6.00)	29 (6.40)	0.93/p=1.00
**TMB≥20 mut/Mb**	5 (0.99)	6812 (9.46)	0.10/p<0.05	2 (1.24)	3 (0.88)	1.42/p=0.96	0 (0.00)	5 (1.28)	0.98/p=1.00	1 (0.98)	4 (1.00)	0.98/p=1.00	2 (4.00)	3 (0.66)	6.25/p=0.38
**N**	**503**	**72003**		161	342		84	300		102	401		50	453	
***MMR***	3 (0.60)	1485 (2.06)	0.00/p<0.05	2 (1.24)	1 (0.29)	4.29/p=0.76	0 (0.00)	3 (0.77)	0.00/p=1.00	0 (0.00)	3 (0.75)	0.00/p=1.00	0 (0.00)	3 (0.66)	0.00/p=1.00
**N**	**250**	**42318**		82	168		50	200		53	197		30	220	
**PD-L1 low positive**	95 (38)	13711 (32.4)	1.28/p=0.10	26 (31.71)	69 (41.07)	0.67/p=0.61	18 (36.00)	77 (38.50)	0.90/p=1.00	22 (41.51)	73 (37.06)	1.21/p=1.00	17 (56.67)	78 (35.45)	2.38/p=0.21
**PD-L1 high positive**	100 (40)	13542 (32)	1.44/p=0.009	46 (56)	54 (32)	2.7/p=0.007	20 (40)	80 (40)	1/p=1	20 (38)	81 (41)	1/p=0.89	3 (10)	97 (44)	0.14/p=0.005
Other signatures															
**N**	**384**	**55023**		129	255		84	300		77	307		37	347	
***HRD* signature positive**	4 (1.04)	2176 (3.95)	0.26/p=0.003	1 (0.78)	3 (1.18)	0.66/p=1.00	1 (1.19)	3 (1.00)	1.19/p=1.00	2 (2.60)	2 (0.65)	4.07/p=0.80	0 (0.00)	4 (1.15)	0.00/p=1.00
**N**	**503**	**72003**		161	342		112	391		102	401		50	453	
***APOBEC***	13 (2.58)	3491 (4.85)	0.52 p=0.03	4 (2.48)	9 (2.63)	0.94/p=1.00	3 (2.68)	10 (2.56)	1.05/p=1.00	2 (1.96)	11 (2.74)	0.71/p=1.00	2 (4.00)	11 (2.43)	1.67/p=0.75
***POLE***	0 (0.00)	47 (0.07)	0.00/p=1.00	0 (0.00)	0 (0.00)	For/p=1.00	0 (0.00)	0 (0.00)	nan/p=1.00	0 (0.00)	0 (0.00)	nan/p=1.00	0 (0.00)	0 (0.00)	nan/p=1.00
***Tobacco signature***	4 (0.80)	7321 (10.17)	0.07/p<0.05	1 (0.62)	3 (0.88)	0.71/p=1.00	1 (0.89)	3 (0.77)	1.17/p=1.00	1 (0.98)	3 (0.75)	1.31/p=1.00	1 (2.00)	3 (0.66)	3.06/p=0.75

Genomic Alterations are in Bold.

This study identified more than 25 different *RET* fusions in the NSCLC cohort. The most frequent alterations accounting for more than 90% of the *RET* fusions included *RET* - *KIF5B* (60.3%), *RET-CCDC6* (17.2%), *RET–RET* (9.6%) and *RET-NCOA4* (3.2%).

We further divided the cohort into histologic subtypes and provided a descriptive analysis of the distribution of the various GAs. **Table 1** shows the distribution of various biomarkers and GA in our cohort along with their distribution based on histologic subtypes of adenocarcinoma. The distribution of the histology revealed that high grade (HG) solid non-acinar 161 (32%) was the most frequent subtype, followed by Undetermined subtype 112 (22.27%), papillary (PAP) 102 (20.28%), and acinar (AC) 50 (9.94%). Other subtypes seen were mucinous/sigmoid (MUCSIG) 49 (9.74%), SCC (2.78%), sarcomatoid (SAR) 8 (1.59%), Lepidic 5 (1%), and SEC 2 (0.4%).

On analyzing potentially targetable GAs, most alterations were found in lower frequency in the *RETfus+* vs *RETfus-* cohort. These included *BRCA1* 1(0.20%) vs 1066 (1.48%) OR 0.13/p<0.05], and *BRAF* [5 (0.99) vs 3864(5.38) OR 0.18/p<0.05]. Others included *FGFR1* [4 (0.80) vs 3189 (4.44) OR 0.17/p<0.05], *KEAP1* [15 (2.98) vs 9711 (13.51) OR 0.20/p<0.05], *KMT2D* [10 (1.99) vs 4690 (6.53) OR 0.29/p<0.05], *KRAS* [12 (2.39) vs 22226 (30.92) OR 0.05/p<0.05], *MDM2* [45 (8.95) vs 3083 (4.29) OR 2.20/p<0.05], *MET* [6 (1.19) vs 3772 (5.25) OR 0.22/p<0.05], *NF1* [10 (1.99) vs 5791 (8.06) OR 0.23/p<0.05], *NSD3* [5 (0.99) vs 3583 (4.98) OR 0.19/p<0.05], *PBRM1* [3 (0.60) vs 1671 (2.32) OR 0.25/p=0.03], *PIK3CA* [13 (2.58) vs 8231 (11.45) OR 0.21/p<0.05], *RB1* [20(3.98) vs 5988 (8.33) OR 0.46/p<0.05], *SMARC4* [10 (1.99) vs 5019 (6.98) OR 0.27/p<0.05], *SOX2* [3 (0.60) vs 4553 (6.33) OR 0.09/p<0.05], *STK11* [12 (2.39) vs 11641 (16.20) OR 0.13/p<0.05], *TERC* [12 (2.39) vs 3357 (4.67) OR 0.50/p=0.05], *TP53* [211 (41.95) vs 50329 (70.02) OR 0.31/p<0.05], and *ZNF703* [6 (1.19) vs 2650 (3.69) OR 0.32/p<0.05]. *SETD2* [54 (10.74) vs 2078 (2.89) OR 4.05/p<0.05] was the only marker that was elevated in *RETfus+* NSCLC.

Signatures were analyzed from a sub-cohort of 384 *RETfus+* and 55023 *RETfus-* NSCLC. All signatures such as *HRD* [4(1.04) vs 2176(3.95) OR 0.26/p<0.05], *APOBEC* [13 (2.58) vs 3491 (4.85) OR 0.52 p=0.03], and *Tobacco* [4 (0.80) vs 7321 (10.17) OR 0.07/p<0.05] were lower in *RETfus+* NSCLC.

The sub cohort studying TMB involved 503 *RETfus+* and 71997 *RETfus-* NSCLC. The median TMB was 2.41 and 6.25 respectively. Both high [32 (6.36) vs 23881 (33.17) OR 0.14/p<0.05] and ultra-high [5 (0.99) vs 6812 (9.46) OR 0.10/p<0.05] were lower in *RETfus+.* However, from a cohort of 250 *RETfus+* and 42318 *RETfus-* NSCLC, High positive PD-L1 [100 (40) vs 13542 (32) OR 1.44/p=0.009] was significantly more in *RETfus+* NSCLC.

On reviewing the distribution of the various GAs *RETfus+* and *RETfus-* NSCLC stratified by histologic subgroups, none of them revealed a statistically significant difference. This data is shown in **Table 1**.

For the *RETfus+* and *EGFR* short variant mutation positive NSCLC cases (28 total cases), the top 7 co-altered genes with both *RET* and *EGFR* were *TP53* (75.9%), *CDKN2A* (37.9%), *MTAP* (27.8%), *NFKBIA* (27.6%), *CDKN2B* (24.1%), *NKX2-1* (24.1%) and *CTNNB1* (20.7%).

## Discussion

From a very large database, we show that advanced NSCLC with *RETfus+* has a unique genomic profile and may represent and unique subtype within NSCLC, both clinically and pathologically. *RETfus+* occurs in less than 2% of all NSCLC. Most studies have found *RETfus+* to occur between 1-2%, which is close to what we found in our analysis (0.69%) (6, 19). From the COSMIC database, *RET* alterations were noted in 4.06% (239/5882) in all lung cancer specimens (20). Since *RET* alterations can occur as acquired resistance to *EGFR* therapy (21), the above analysis excluded patients with *EGFR* alteration, which improves the validity of our analysis.

*RETfus+* NSCLC in our analysis was found to have significantly lesser frequency of most targetable and un-targetable GAs. This is likely because *RET* fusion is the primary driver in these cases. However, understanding the molecular spectrum is important to understand treatment strategies for when the disease progresses on *RET* inhibitors, as well as for developing further targeted agents (22). Understanding the mutational signatures is also important to analyzing the mechanism and development of resistance to *RET* directed therapy (23). There are around 45 *RET* fusions with various partners, with *KIF5B-RET* being the most common. This may have treatment implications as *RET* inhibitors like Vandetanib and Pralsetinib may show a difference in response based on the type of fusion partner (24–26). Several mechanisms of resistance, both intrinsic and acquired have been described. *MET* amplification is one of the most reported mechanisms of resistance to *RET* inhibitors (27). *MET* amplification can occur even after modest treatment periods with *RET* inhibitors like selpercatinib, showing that mutational signatures can change with the clinical course. *RET* alterations can disappear and re-appear as the clinical course fluxes between response and progressive disease (28). Acquired resistance to *RET* via *MET* amplification can be overcome by combining *RET* inhibitors with *MET* directed therapy like crizotinib. Limited anecdotal evidence for this is available from preclinical studies and small case series. *MET* alteration may also occur as an intrinsic mutation causing primary resistance (27). Only 6 patients with *MET* GA in our relatively large *RETfus+* cohort were found, highlighting the challenge faced in studying this phenomenon. Other *MAPK*-activating changes have been reported as a cause of acquired resistance. These include *KRAS, BRAF* and *FGFR1*, where were observed at a frequency of 2.39%, 1% and 0.8% in our *RETfus+* cohort respectively. It can be hypothesized that *RET* inhibitors like Selpercatinib may eliminate the *RET* positive cells and create a selective pressure enabling cells with other *MAPK* alteration to proliferate (5). Primary resistance may be caused by alterations in *PI3K* pathway such as *PIK3CA* (2.58% in our cohort) or *PTEN* mutations. GA in *MAPK* pathway like *KRAS* alleles can also cause primary resistance (5). *SMARCA4* (2% in our cohort) is another GA associated with primary *RET* resistance (29). Besides these, several gatekeeper mutations of *RET* itself result in both primary and secondary resistance (22). *RET* alteration is an important mechanism of acquired resistance to *EGFR* TKIs including osimertinib (30). When these co-exist, studies have hypothesized and demonstrated re-sensitization of malignant cells to *EGFR* inhibition when combined with *RET* inhibition. A similar strategy could potentially be evaluated when resistance develops to *RET* inhibitors, but very little data is available on the same (31). *SETD2* (SET domain containing 2, histone lysine methyltransferase) occurs in about 7% of all NSCLC (32). In our *RETfus+* cohort, this occurred at around 11%. *SETD2* is involved in DNA methylation and DNA repair through depletion of its product, histone H3 lysine 36 (*H3K36*) trimethylase. These sensitize them to Hypomethylating agents which has been demonstrated in studies utilizing cell lines. Combining hypomethylating agents and *PARP* inhibitors may be a strategy that can be explored in the future (33).

Several advancements have been made in the past decade in the therapeutic spectrum of *RETfus+* NSCLC (34). Initial trials investigated multikinase inhibitors like Cabozantinib, Lenvatinib and Vandetinib. Cabozantinib had response rates ranging between 28-50%, Vandetinib 18-47% and Lenvatinib at 16% (34). Other agents like sunitinib, sorafenib, alectinib, nintedanib, ponatinib, and regorafenib had modest responses, and were not extensively studied (34). It was only a matter of time until new selective *RET* inhibitors made its way, which started with the LIBRETTO-001 trial (35). 105 patients with platinum refractory or relapsed *RETfus+* NSCLC were enrolled and treated with Selpercatinib. The objective response rate (ORR) was 64%. In the untreated setting, the ORR was 85% among 39 patients. Among 11 patients with CNS disease the ORR was 91% (6). Similar results, but with smaller numbers were noted with Pralisetinib (7). The NCCN guidelines recommend selpercatinib, Pralesertinib and Cabozantinib as first line options for *RETfus+* NSCLC (36). Pemetrexed based chemotherapy remains the standard second line option but results with immune checkpoint therapy have yielded underwhelming results. Results on checkpoint therapy use in this setting from real-word and retrospective analysis have shown a varying result, but the consensus remains that it does not work, due to the “biologically cold” nature of *RETfus+* tumors (22). Checkpoint markers in our analysis have shown conflicting results with PD-L1 high seen more and high/ultra-high TMB seen in lesser frequency in the *RETfus+* cohort. *STK11* was also less often seen in the *RETfus+* group. These data support the hypothesis that checkpoint therapy is not efficacious in mutationally driven NSCLC and may even be detrimental if started early by augmenting toxicities like pneumonitis (37).

Lung adenocarcinoma has distinct histologic subtypes and each subtype carry unique genomic and prognostic significance (38). From our study, HG and PAP were noted more frequently in *RETfus+* NSCLC. Both are high-grade variants and may be representative of aggressive disease with poor prognosis (39, 40).

Other studies have analyzed the genomic characteristics of *RETfus+* NSCLC. A study on Chinese NSCLC patients showed that *RETfus+* was noted 1.43% of 174 and *KIF5B-RET* was the most common fusion partner (41). Another study reported low frequencies of co-existing alterations like *EGFR, KRAS* and *ALK* (42). But our analysis gives a descriptive overview of other GAs that co-occur in *RETfus+* NSCLC, from a relatively large cohort, which may help understand the disease biology and support future studies. Our study does carry limitations due to the lack of correlating clinical outcome data. Regardless, understanding the GA of distinct molecular subtypes of NSCLC is important as evidenced by our prior work along the same lines (11).

In summary, *RETfus+* NSCLC represents a relatively rare genomic category of NSCLC that is usually devoid of other alterations. Although targetable with oral agents (6), more research is needed to determine the best beyond first line treatment options. In addition, these findings are important as a guide to increasing accuracy in genomic testing especially for small sample sizes such as fine-needle aspiration biopsies and, in the future, for patient who have had liquid biopsies that feature a low tumor fraction. The combination of histologic and genomic features can help determine if *RETfus+* has been falsely negative in specimens constrained by tumor cell availability.

## Data Availability

The original contributions presented in the study are included in the article/supplementary material. Further inquiries can be directed to the corresponding author.
